# The study of miRNA-211 affects melanogenesis progress in Cashmere goats via suppressing *AP1S2*

**DOI:** 10.5194/aab-68-125-2025

**Published:** 2025-02-11

**Authors:** Baoyu Zhang, Runlai Liu, Tao Ma, Xinyu Li, Yuxin Zhao, Huixian Su, Jianping Li, Yumei Li, Huaizhi Jiang, Qiaoling Zhang

**Affiliations:** 1 College of Veterinary Medicine, Jilin University, Changchun 130062, China; 2 College of Animal Science and Technology, Jilin Agricultural Science and Technology University, Jilin 132109, China; 3 College of Animal Science, Jilin University, Changchun 130062, China; 4 College of Animal Science and Technology, Jilin Agricultural University, Changchun 130033, China

## Abstract

Chromaticity is a key indicator of measuring economic worth of Cashmere goats, and gene regulation plays a major role in Cashmere goat color. By targeting the 3^′^UTR region of genes, such as miRNA-211, miRNA plays a significant regulatory role in melanogenesis. But the mechanism of how miRNA-211 affects the melanogenesis in Cashmere goats is unclear. As a result, insights into the color regulation by miRNA-211 in Cashmere goats and its brief mechanism are offered in this study. First, the target gene *AP1S2* of miRNA-211 was screened by TargetScan and GO/KEGG, and then the targeted relationship was confirmed by the dual-luciferase reporter experiment. In B16-F10 cells, which overexpress miRNA-211 and goat skin, the opposite expression trends between *AP1S2* and miRNA-211 were analyzed by reverse transcription–quantitative PCR (RT-qPCR) and western blot. And their opposite expression trends were confirmed in B16-F10 cells by immunofluorescence. The information above showed miRNA-211 downregulated the expression of *AP1S2*. Afterwards, the impact of miRNA-211 targeting *AP1S2* on the melanogenesis of Cashmere goats was confirmed by animal regression studies. Utilizing immunohistochemistry, the expression of *AP1S2* in a mouse hair follicle was observed. Following subcutaneous injection of antagomiRNA-211 (chemical modification inhibitor), the expression levels of the *AP1S2* gene and protein were enhanced, and the localization in the hair follicle was also increased. Furthermore, reduction in melanin content in the skin was detected. These results showed that miRNA-211 significantly affected the melanogenesis progress via downregulating *AP1S2* and advantageously affected the melanin content of Cashmere goat skin.

## 1 Introduction

The deposition of mixed pigments in keratinocytes determines the color phenotype of skin and hair in humans and animals (Pavan and Sturm, 2019). Melanin is a phenolic macromolecular polymer generated in organelles called melanosomes and transported to certain epidermal keratinocytes or hair cortical medullary layers for deposition (Hushcha et al., 2021; Weiner et al., 2014). With the deepening of research, miRNA has been found to play an increasing number of roles in biological functions such as immunity, metabolism, cellular communication, and melanin deposition. Factors such as UVB and miRNA released by keratinocytes usually influence the production and transportation of melanin during pigmentation (Domingues et al., 2020). The expression of the melanocyte inducing transcription factor (MITF), a crucial transcription factor in melanin synthesis, is inhibited by miRNA such as miR-125b-5p, which affects the biological activity and melanin synthesis of melanocytes in vitiligo patients (Wang et al., 2022). Recent research had demonstrated that the protein expression levels of SOX5, β -catenin, and CDK2 were downregulated by miR-21-5p, which eventually stimulated melanogenesis, reducing pigmentation in vitiligo patients (Aguennouz et al., 2021). And the research team previously discovered that miRNA-202, miRNA-200a, and miRNA-193b also played a role in determining the coat color of Cashmere goats by regulating their target genes (Qu et al., 2017; Li et al., 2022; Xiang et al., 2023).

*AP1S2* encodes the subunit of adapter protein complex 1 (AP-1), which plays an important role in the assembly of grid proteins. Previous studies demonstrated that *AP1S2* was associated with the occurrence of related diseases, such as ovarian cancer, Alzheimer's disease, and hydrocephalus, serving as a biomarker and a new therapeutic target. *AP1S2* is involved in the metastasis and dissemination of cancer cells. After silencing the expression of *AP1S2* in A375 cells, the migration capacity of melanoma cells was significantly reduced (Margue et al., 2013). At the same time, *AP1S2*, as the main target gene of miRNA-204-5p and miRNA-211-5p, was involved in the process of melanoma migration and melanogenesis. LINC00518, as a long non-coding RNA (lncRNA), inhibited miR-204-5p expression and upregulated the expression of *AP1S2* to promote the metastasis of malignant melanoma (Luan et al., 2019). Moreover, AP-1 has been proven to control the recruitment of grid proteins and is a key protein for ATP7A transport, ATP7A was an important protein that provides copper for tyrosinase. Due to AP-1 silencing, ATP7A could not be transported to the trans-Golgi network (TGN), affecting the transport of copper from ATP7A to melanosomes (Holloway et al., 2013). It is thus clear that *AP1S2* (AP-1 subunit encoding gene) is closely related to the process of melanin production.

Both miRNA-204 and miRNA-211 belong to the miRNA-204 family and are situated on *TRPM1*'s intron and co-expressed with host genes (Vitiello et al., 2017). Mazar et al. (2010) discovered that the expression of miRNA-211 is dependent on MITF activity and plays an important regulatory role in human melanoma. When UVB radiation damaged the DNA, the *MALAT1*-miRNA-211-*SIRT1* axis was activated in the diseased skin tissue of vitiligo patients, and miRNA-211 was significantly downregulated, decreasing the possibility of skin cancer in the afflicted area (Brahmbhatt et al., 2021). The study in 2020 indicated that the expression of miRNA-211 was reduced under UVB stimulation, raising p53 and MMP9 expression in melanocytes. The pigmentation might be improved by restricting the expression of MMP9 in vitiligo patients' skin (Su et al., 2020). According to research of Tian et al. (2012), high-throughput sequencing technology also revealed that the expression of some miRNA, including miRNA-211, varied in the skin tissues of alpacas with varying fur colors, whereas it was unclear how miRNA-211 affected pigmentation. Therefore, this study explores the mechanism of miRNA-211 in the process of melanogenesis in Cashmere goat skin in vitro and in vivo. The regulation on *AP1S2* by miRNA-211 enriched people's understanding of the process of melanogenesis, and the advantageous effect of miRNA-211 on melanin content would provide a theoretical basis for artificial regulation of coat color.

## 2 Materials and methods

### 2.1 Animal sample collection

The study received approval from Jilin University's Experimental Animal Welfare and Ethics Committee. The skin of all male adult Cashmere goats (four white Cashmere goats and four black Cashmere goats) was sourced from the Baicheng Breeding Center of Jilin Province, and all Cashmere goats were raised under the same feeding conditions. The skin samples were collected and quickly placed in a liquid nitrogen tank for freezing and then transferred to - 80 °C conditions for storage. All 20 d old female BALB/c mice from Liaoning Changsheng Biotechnology were subcutaneously injected with 0.5 OD antagomiRNA-211 and phosphate buffer saline (PBS), respectively, for 7 consecutive days in the experimental group and the control group. After euthanasia, skin tissue was taken, part of the skin was soaked in 4 % paraformaldehyde, and the rest of the skin was frozen at - 80 °C. Part of the skin was soaked in 4 % paraformaldehyde, and the other part of the skin was frozen at - 80 °C.

### 2.2 Target gene prediction and screening

Target genes' information of miR-211-5p were obtained through miRBase (https://www.mirbase.org/, last access: 24 June 2013) and TargetScanHuman 8.0 (https://www.targetscan.org/vert_80/, last access: September 2021) bioinformatics online websites. Gene IDs were obtained via the DAVID (https://david.ncifcrf.gov/home.jsp) website. GO/KEGG enrichment analysis was utilized by KOBAS 3.0 (http://bioinfo.org/kobas/, last access: December 2019) and Metascape (https://metascape.org/gp/index.html#/main/step1, last access: 18 December 2021), which analyzed the biological functions and screened genes related to the pigment synthesis pathway. Reverse transcription–quantitative PCR (RT-qPCR) primers for the target genes (Table 1) and miRNA-211 (Table 2) were synthesized by Comate Bioscience Co., Ltd. (Changchun, China).

**Table 1:** Primer sequences of quantitative real-time PCR for target genes.

Gene	Primer	Primer sequence (5^′^–3^′^)
*AP1S2*	F	AATTGAGCAGGCCGATCTCC
(*Capra hircus*)	R	GACATGAGAGGCAGTGACCA
*AP1S2*	F	AATTGAGCAGGCCGATCTCC
(*Mus musculus*)	R	CATCAGCAAGGGAGGACAGTT
*GAPDH*	F	CCGTAACTTCTGTGCTGTGCC
(*Capra hircus*)	R	TGAAGGGGTCATTGATGGCAAC
*GAPDH*	F	AAGAGGGATGCTGCCCTTAC
(*Mus musculus*)	R	GTTCACACCGACCTTCACCA

F: forward primer. R: reverse primer.

**Table 2:** Primer sequences of real-time PCR for miR-211.

Gene	Primer	Primer sequence (5^′^–3^′^)
miRNA-211	RT	GTCGTATCCAGTGCAGGGTCCGAGGTATTCGCACTGGATACGACAGGCAA
	F	CGCGTTCCCTTTGTCATCCT
	R	AGTGCAGGGTCCGAGGTATT
U6	RT	CGCTTCACGAATTTGCGTGTCAT
	F	GCTTCGGCAGCACATATACTAAAAT
	R	CGCTTCACGAATTTGCGTGTCAT

F: forward primer. R: reverse primer. RT: stem-loop RT primer.

### 2.3 Construction of plasmids and dual-luciferase assay

To verify *AP1S2* was targeted by miRNA-211, the psiCHECK-2 plasmid, which had been preserved in DH5 α strain from our lab, was isolated using a plasmid extraction kit (TIANGEN, Beijing, China). Target gene segments with constrained sticky ends were generated by the annealing connection. Primers were designed as in Table 3. The wild-type seed sequence was transformed into a mutation-type one to avoid the stop code mutation, utilizing the site-directed mutagenesis technology. The 3^′^UTR region seed sequences of psi-*AP1S2* (control), wt-*AP1S2* (wild-type) and mut-*AP1S2* (mutation-type) were amplified by PCR. Subsequently, the amplified fragments were inserted between the *Xho*I and *Not*I (Takara, Liaoning, China) restriction endonuclease sites of psiCHECK-2. After transducing the DH5 α bacterial community and conducting positive screening and bacterial liquid sequencing, the reconstructed plasmid was extracted using an endotoxin-free plasmid extraction kit (Omega, Beijing, China). HEK-293T cells were seeded in 96-well plates for a transfection experiment, with $5\times 10^{5}$ cells per well. HEK-293T cells were transfected with Lipofectamine 3000 (Thermo Fisher Scientific, Shanghai, China) containing miRNA-211 mimics (mi) and negative control (nc), respectively. After culturing for 24 h, the cell lysate was used to detect luciferase activity using the Dual-Luciferase Reporter Assay (Promega, Beijing, China).

**Table 3:** Primers of target gene annealing.

Gene	Primer	Primer sequence (5^′^–3^′^)
*AP1S2*	wt-F	TCGATACCCAATTTTTTTTAAAGGGAACTTTGGTTAATCAAA
	wt-R	GGCCTTTGATTAACCAAAGTTCCCTTTAAAAAAAATTGGGTA
	mut-F	TCGATACCCAATTTTTTTTAATGGTAACTTTGGTTAATCAAA
	mut-R	GGCCTTTGATTAACCAAAGTTACCATTAAAAAAAATTGGGTA

F: forward primer. R: reverse primer. wt: wild type. mut: mutation type.

### 2.4 Reverse transcription–quantitative PCR (RT-qPCR)

In this study, RNA was extracted from animal skin and cells using TRIgent reagent (Mei5bio, Beijing, China). The reverse transcription kit (Innovagene, Changsha, China) was utilized to generate the cDNA of the target gene and miRNA. According to the real-time fluorescence quantitative PCR kit (Innovagene, Changsha, China), the experimental apparatus was configured. The initial process was pre-denaturation for 2 min at 94 °C. After that, there were 40 cycles of denaturation for 15 s at 94 °C, annealing for 40 s at 55 °C, and extension for 25 s at 72 °C. Finally, the experiment was terminated using the disintegration conditions of the system itself. The relative expression level of the PCR amplification products is analyzed using the Livak technique. Specifically, the average internal reference CT value was determined for every group, and the average internal reference CT value was removed from the target gene CT value to determine the Δ CT value. To calculate ΔΔ CT, the mean Δ CT value of the control group was subtracted from the Δ CT values of the experimental and control groups. Lastly, the expression differences between the two groups were compared using $2^{-\Delta \Delta \mathrm{CT}}$ .

### 2.5 Western blot analysis

The skin tissue samples of Cashmere goats and B16-F10 cells were placed in the RIPA (BioWorld, USA) extraction lysate buffer containing phenylmethylsulfonyl fluoride (PMSF). The total protein concentrations in each group were quantified using the BCA protein assay kit (Solarbio, Beijing, China). The protein was transferred to a polyvinylidene fluoride (PVDF) membrane (Bio-Rad Laboratories, Inc.). And the PVDF membrane was incubated with the Rabbit Anti-*AP1S2* Antibody ( 1:500 ; GeneTex, USA), a product of *AP1S2* (GeneTex) with strips located between 17kDa and 26kDa (Fig. S1a in the Supplement); β -Actin Monoclonal antibody ( 1:5000 ; Proteintech, Wuhan, China), with strips located between 45kDa and 75kDa; and a secondary antibody named horseradish peroxidase (HRP)-conjugated AffiniPure Goat Anti-Mouse IgG (H + L) (Boster, Wuhan, China). Protein expressions were analyzed using an enhanced chemiluminescence (ECL) detection kit (Invitrogen, Shanghai, China). Images with the proper signal strength under various gradient exposure durations were screened using the fully automated chemiluminescence image analysis system (Tanon). ImageJ software was utilized to quantify the strips in grayscale and then calculated the target strips' relative grayscale value. The β -actin protein bands in this experiment are shown in Fig. S2.

### 2.6 Transient overexpression

B16-F10 (MeilunBio, Liaoning, China), a clonal subline of B16 cells from C57BL/6J epithelial melanoma tissue, was cultivated in a six-well plate with $10^{6}$ cells per well for 24 h before transfection to overexpress miRNA-211. Negative controls (nc), miRNA-211 mimics (mi), and miRNA-211 inhibitors (ih) were transfected to cells using Lipofectamine 3000 once the cell density reached 70 %–90 %. RNA was extracted at 24 h and protein was extracted at 48 h for analyzing the expression of *AP1S2* after the transient transfection.

### 2.7 Immunofluorescence

After 24 h of transfection, cells were treated with 4 % paraformaldehyde for 15 min. After penetrating for 30 min with 0.3 % Triton X-100, cells were blocked for 1 to 2 h with 3 % bovine serum albumin (BSA). The cells were then incubated in a damp box with the anti-*AP1S2* antibody ( 1:500 ; GeneTex, USA) at 4 °C for the whole night. The Goat Anti-Mouse IgG H&L (fluorescein isothiocyanate, FITC) (Abcam, Shanghai, China) was added to cells at 20 °C and incubated for 2 h. The cells were then stained by DAPI for 5 min. An antifade mounting medium (Biyotime, Beijing, China) was used for sealing the film. The fluorescent microscope was utilized for imaging and recording the expression of *AP1S2*. ImageJ was used to convert monochrome fluorescent images to grayscale images, substitute grayscale values for fluorescence intensity, and then divide the total fluorescence intensity (IntDen) by the region's area (Area) to determine the average fluorescence intensity.

### 2.8 Immunohistochemistry

The skin tissues of mice in the antagomiRNA-211 and PBS groups were set in 4 % paraformaldehyde. Sections of the paraffin-embedded tissue were sliced into sections that were 4–6 µM by the slicer and placed on a glass plate. Slices were penetrated by 0.3 % Triton X-100 and were then blocked for 2 h by 3 % BSA. The slices were then incubated by the anti-*AP1S2* antibody ( 1:500 ; GeneTex, USA) for the whole night at 4 °C in a wet box and then washed by tris-buffered saline (TBST). And then slices were incubated by the goat anti-mouse secondary antibody at room temperature for 30 min and washed by TBST. Once a suitable brown precipitate had formed, the color reaction with DAB substrate was hindered and slices were rinsed with water and sealed with gum. The optical microscope was utilized to determine the expression of *AP1S2*. To identify high-positive, positive, low-positive, and negative results, the staining area and intensity of the positive cells are scored using ImageJ's IHC Profiler plugin.

### 2.9 The extraction and determination of melanin

The skin tissues of mice in the antagomiRNA-211 and PBS groups were broken into powders after being frozen in liquid nitrogen. The powders were then hydrolyzed for 16 h at 55 °C with papain (20 mg mL^−1^) and then filtered using gauze. The supernatant was disposed of following centrifugation for 10 min at 3500 rpm. The precipitate was cleaned with petroleum ether, anhydrous ethanol, and distilled water until the PH was neutral. Then it was diluted by 0.1 mol L^−1^ NaOH to 200 µL at the 96-well plate after freeze drying. A wavelength of 500 nm was used to measure the absorbance.

### 2.10 Statistical analysis

The data in this study were expressed as the means ± standard deviations (SDs) of three separate experiments and were analyzed using one-way ANOVA and two-tailed Student's t test in SPSS 13.0. The results were visualized and analyzed using GraphPad Prism. Four symbols denote statistical significance: ^*^ denotes a significance level of p< 0.05, ^**^ of p< 0.01, ^***^ of p< 0.001, and ^****^ of p< 0.0001.

## 3 Results

### 3.1 Screening of miRNA-211 target genes

An online bioinformatics website was used to screen for genes that interact well with miRNA-211-5p. GO and KEGG enrichment analysis revealed that the target genes were involved in melanogenesis biological processes such as phosphorylation and mitogen-activated protein kinase (MAPK) cascade reactions. Additionally, *AP1S2*, a gene related to endocytosis and protein transport processes, was also screened. *AP1S2* is involved in the transport of substances within lysosomes and has been shown to be involved in the transport of important cargo proteins within melanocytes. Therefore, it was chosen as the research object of this experiment.

### 3.2 Confirmation of interaction between miRNA-211 and *AP1S2*

**Figure 1:**
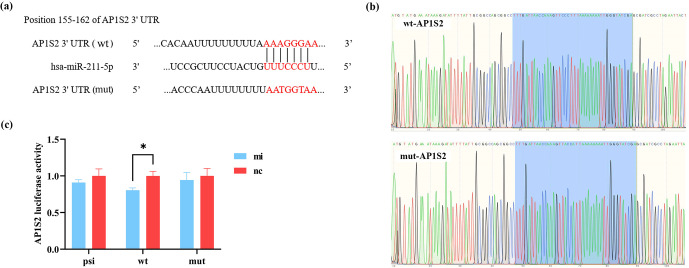
miRNA-211 targets on *AP1S2*. **(a)** The binding sites of wild-type (wt) and mutation-type (mut) target gene and miRNA-211-5p. **(b)** The sequencing results of the recombinant plasmid (marked in blue) were consistent with the wt and mut sequences of the target gene. **(c)** The validation results of the dual-luciferase reporting system showed that the expression of psi-*AP1S2*, wt-*AP1S2*, and mut-*AP1S2* after transfecting HEK-293T cells with miRNA-211 mimics (mi) and negative control (nc) (^*^ denotes p < 0.05). psi: psiCHECK-2 (control type), mut: mutation type, wt: wild type.

The binding sites of miRNA-211 with wild-type and mutation-type target genes are shown in Fig. 1a. The sequencing map identified that the vector was constructed successfully. The accurate presentation of wild-type and mutation-type sequences of the target gene in the bacterial liquid sequencing map indicated the successful construction of the reconstructed vector (Fig. 1b). The results of the dual-luciferase reporter system showed that miRNA-211 significantly affected the expression of *AP1S2*. Compared to the negative control (nc), the fluorescence of the wild type in the miRNA-211 mimics (mi) group decreased significantly, while the fluorescence of the mutation type did not change in each group (Fig. 1c); the result suggested that miRNA-211 interacted with the 3^′^UTR region of *AP1S2* and that the predicted binding site was AAAGGGAA.

### 3.3 The contrary expression levels of miRNA-211 and *AP1S2* in skin tissues of Cashmere goats

**Figure 2:**
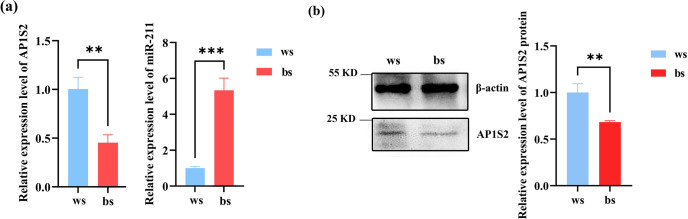
The target gene and miRNA-211 expressed conversely in white (ws) and black (bs) skin tissues of Cashmere goats. **(a)** The relative mRNA expression of miRNA-211 and *AP1S2* in white and black skin tissues (^**^ denotes a p < 0.01 and ^***^ a p < 0.001). **(b)** The relative protein expression of *AP1S2* in white and black skin tissues (^**^ meaning p < 0.01).

The results of RT-qPCR and western blot analysis showed that miRNA-211 might downregulate the expression of *AP1S2* in skin tissues. In contrast to the black skin tissues of Cashmere goats, the relative mRNA level of *AP1S2* in white tissues was significantly higher, whereas the expression of miRNA-211 was notably lower (Fig. 2a), the results indicated that *AP1S2* might be negatively regulated by miRNA-211. The results of western blot showed that relative protein expressions of *AP1S2* showed the same trend as mRNA in different tissues (Figs. 2b, S1d). In conclusion, miRNA-211 might suppress the expression of *AP1S2* at both mRNA and protein levels.

### 3.4 The expression of *AP1S2* was upregulated after the inhibition of miRNA-211 in B16-F10

**Figure 3:**
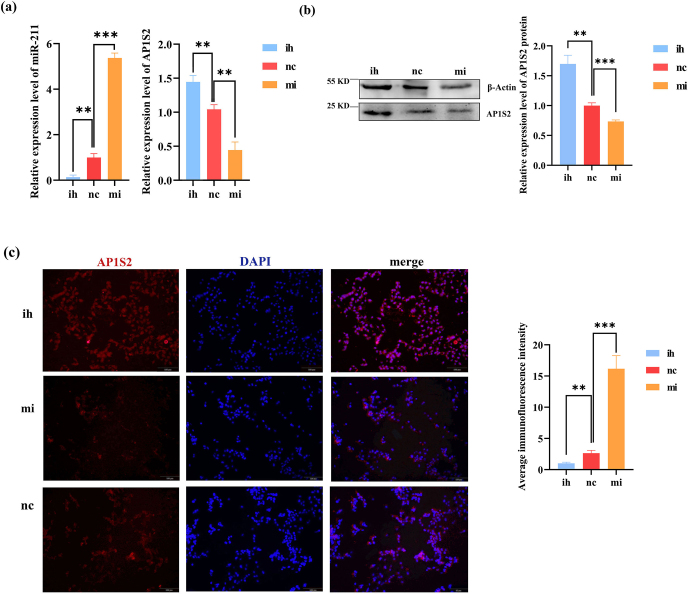
The results show the opposite expressions of miRNA-211 and *AP1S2* after transfecting B16-F10. **(a)** The image on the left shows that miRNA-211 expressed significantly more strongly in the miRNA-211 (mi) group than the miRNA-211 (ih) and (nc) groups. The relative mRNA expression levels of *AP1S2* in miRNA-211 (mi), (nc), and (ih) groups are shown in the image on the right. The expression of *AP1S2* in the miRNA-211 (mi) group showed a decreasing trend in contrast to miRNA-211 (ih) and (nc) groups (^**^ denotes a significance level of p < 0.01 and ^***^ of p < 0.001). **(b)** The relative expression of the *AP1S2* protein in miRNA-211 (mi), (nc), and (ih) groups (^**^ denotes p < 0.01, and ^***^ denotes p < 0.001). **(c)** *AP1S2* (red) was expressed mores strongly in the miRNA-211 (ih) group than in the other two groups and was expressed in both the cytoplasm and the nucleus (blue) of B16-F10 cells. The quantification of relative fluorescence intensity is shown on the right (^**^ denotes p < 0.01, and ^***^ denotes p < 0.001). mi: mimics, nc: negative control, ih: inhibitors.

The results of the overexpression experiment revealed that the expression of *AP1S2* in B16-F10 was negatively regulated by miRNA-211. Compared to the inhibitor group (ih) and the negative control group (nc), the expression of miRNA-211 was significantly increased in the miRNA-211 mimics group. Conversely, the expression of *AP1S2* decreased at the mRNA and protein levels (Figs. 3a, b and S1b, c). The immunofluorescence experiment's findings demonstrated that *AP1S2* was expressed in both the cytoplasm and the nucleus of B16-F10 cells. After transfection with B16 cells, the fluorescence signal intensity of *AP1S2* in the miRNA-211 mimics group was significantly lower than that in the inhibitor group (Fig. 3c). These results indicate that *AP1S2* was negatively regulated by miRNA-211.

### 3.5 Verification of the impact of miRNA-211 on *AP1S2* by animal experiments

**Figure 4:**
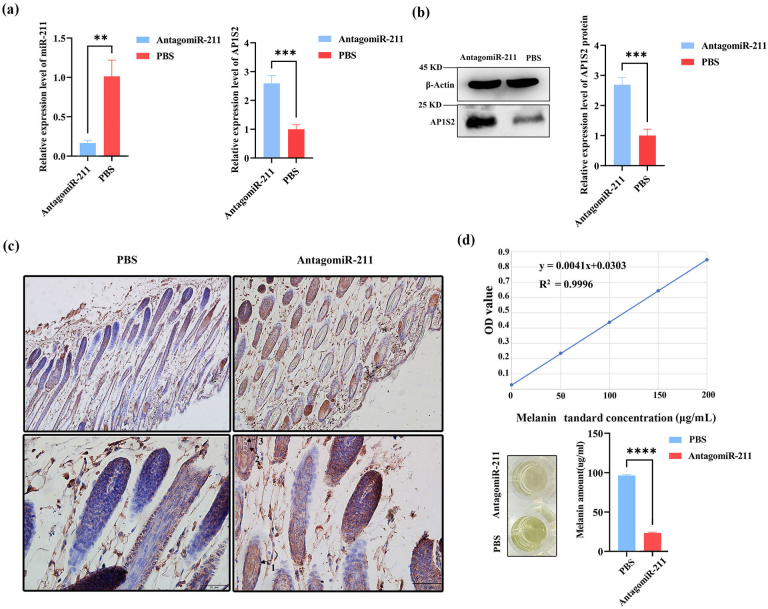
The expression of *AP1S2* and pigment content in the skin of mice injected with antagomiRNA-211. **(a)** The relative mRNA expressions of miRNA-211 and *AP1S2* in the antagomiRNA-211 group and the PBS group. (^**^ denotes a significance level of p < 0.01 and ^***^ of p < 0.001). **(b)** The relative protein levels of *AP1S2* in the antagomiRNA-211 group and the PBS group (^***^ denotes p < 0.001). **(c)** *AP1S2* was expressed more strongly in the skin of PBS group than in the antagomiRNA-211 group; arrows 1, 2, and 3 represent the outer root sheath, inner root sheath, medullary layer, and cortical layer, respectively. **(d)** The standard curve of melanin is shown in the top image. Compared with the PBS group, the pigment content was decreased in the antagomiRNA-211 group (^****^ denotes p < 0.0001).

Following subcutaneous injection of antagomiRNA-211 into BALB/c mice, compared with the PBS injection group, the expression level of miRNA-211 was significantly reduced in the antagomiRNA-211 group, while the levels of *AP1S2* protein and mRNA were significantly increased (Figs. 4a, b and S1e, f). The immunohistochemical results showed that *AP1S2* was expressed in the hair follicle shaft, inner root sheath, outer root sheath, and connective tissue (Fig. 4c). The antagomiRNA-211 group was better than the PBS group in terms of cell-positive staining, with a software score that is positive, but the PBS group received a negative score. Compared with the injection of PBS, a considerable decrease in melanin levels was detected after the injection of antagomiRNA-211 (Fig. 4d). The above data indicated that *AP1S2* was negatively regulated by miRNA-211 during pigment generation, and an increase in miRNA-211 expression was beneficial for pigment generation.

## 4 Discussion

The color of animal hair is determined by the type and distribution of melanin, which is mainly divided into true melanin and brown melanin. It originates from pigment cells in the basal layer of the epidermis. True melanin is brown and black in color, and tyrosine and phenylalanine are used as raw materials for its generation. Dobaquinone and cysteine participate in the production of brown melanin precursor substances, ultimately producing yellow and brown products. The generated melanin is transported by actin and microtubules, moving along the melanocyte cytoskeleton or transferring to keratinocytes (D'Alba and Shawkey, 2019). *SLA2*-a/*MLPH* is believed to inhibit the motor protein function by phosphorylating cAMP and protein kinase A (PKA), which is one of the mechanisms leading to retrograde transport of melanosomes along microtubules. In the pigmentation of animal hair, melanocytes in hair follicles send dendrites vertically upwards and provide pigment to two rows of epithelial cells (cortical cells and medullary cells). Epithelial cells generally emit diffusion signals to induce melanocytes to migrate towards them and extend dendrites. In addition, melanocytes are prevented from connecting with adjacent cells of the target cell as adjacent cells cannot emit pigment receptor signals, thereby blocking or repelling pigment deposition. This mechanism achieves precise targeting of pigments (Ohbayashi and Fukuda, 2020).

Melanogenesis is often influenced by a complex regulatory network mediated by miRNA, which targets related genes to activate or inhibit related pathways in melanocytes. At the same time, the activity of miRNA is also regulated by related protein and lncRNA. Chen et al. (2024) demonstrated that the regulatory network composed of mRNA, such as efu-miR-101, mle-bantam-3p, egr-miR-9-5p, and sma-miR-75p, and lncRNA played a crucial regulatory role in the formation of the Manila clam shell color. Dicer-miR92b-ItgaV has been shown to be the main targeted axis for stress-induced hair whitening (Bertrand et al., 2024). miRNA acts on melanocytes through major signaling pathways, including the cAMP-dependent signaling pathway, WNT/ β -catenin signaling pathway, and extracellular regulated protein kinase (ERK) signaling pathway, which ultimately regulate the transcriptional activity of melanocyte inducing transcription factor (MITF), thereby affecting the synthesis of *TYR*, which is a key enzyme in melanin synthesis. MC1R activated the cAMP signaling pathway upon binding to melanocortin, promoting the proliferation of melanocytes (Chen et al., 2017). The production of TYR regulated by MITF was enhanced by activating of canonical Wnt/ β -catenin pathway in the skin of dark *Pletropomus leopardus* (Song et al., 2022). Studies had observed that the melanin content was significantly reduced by the inhibition of MITF in B16-F10, which was induced by miR-181a-5p and miR-199a in the exosomes of human amniotic mesenchymal stem cells (hAMSCs) (Wang et al., 2021). In our previous studies, we confirmed that miR-27, miR-202, miR-200a, and miR-193b negatively regulate corresponding target genes related to melanin production located in the canonical Wnt/ β -catenin pathway, affecting the formation of animal hair color (Qu et al., 2017; Wu et al., 2021; Li et al., 2022; Xiang et al., 2023).

Solexa sequencing in 2012 found that 214 miRNA, including miRNA-211, were differently expressed in white and brown alpaca skin (Tian et al., 2012). In our research miRNA-211 was also verified to express differently in the skin of Cashmere goats with different coat colors. In order to further explore the mechanism of miRNA-211 regulating coat color, we first screened the target genes of miRNA-211 and verified the targeting relationship between miRNA-211 and the target genes and the correlation with coat color in vivo and vitro experiments. In vitro, we selected B16-F10 melanoma cells (retaining the ability of melanocytes to produce melanin and the expression of related genes) as substitutes to melanocytes, and miRNA-211 mimics were transfected in B16-F10 to explore the regulatory relationship between miRNA-211 and *AP1S2* in melanocytes. Ultimately, we found that *AP1S2* was negatively regulated by miRNA-211 in both B16-F10 cells and the skin of Cashmere goats. The study also found that miRNA-211 inhibition reduced melanin content, which provided inspiration for the coat color control of Cashmere goats. miRNA-211 did not only affect animal pigmentation. Dai et al. (2015) also showed that miRNA-211 regulated the migration and pigmentation of melanoma cells through the miRNA-211/*TGF*- β /*TYR* axis. After the transfection of pre-miRNA-211 in melb-a and melan-a cells, the expression of the TGF- β signaling factor in the melanin synthesis pathway was inhibited, ultimately leading to an increase in the expression of *TYR* and *TYRP1*. In addition, the activity of miRNA-211 was induced by MITF, which inhibited the resistance of melanoma cells to BRAFV600E inhibitors and led to an adaptive increase in melanin content when miRNA-211 expression was increased (Vitiello et al., 2017).

AP (adaptor protein) participates in the assembly of clathrin derived from TGN and the plasma membrane, including AP-1, AP-2, AP-3, and M6P. *AP1S2* is one of the subunits of AP-1, and it has been experimentally confirmed that epithelial mesenchymal transition (EMT) activated the transcription factor *ZEB1* to enhance the action of *AP1S2* and accelerate endocytosis and vesicular transport (Banerjee et al., 2021). It was reported that the downregulation of *AP1S2* by miRNA-211 might affect the ability of ATP7A to transport copper, but the suppressed expression of the target gene *EDEM1* ultimately prevented the degradation of TYR and promoted the accumulation of melanin (Vitiello et al., 2017). This explained the significant decrease in melanin content in the skin of BALB/c mice injected with antagomiRNA-211 in this study. Therefore, this experiment speculates that overexpression of miR-211 affects melanogenesis progress by downregulating *AP1S2*, which might affect the transport of ATP7A. However, it does not affect the accumulation of melanin in animal skin.

## 5 Conclusions

In summary, this study suggested that miRNA-211 affected the process of melanin production by regulating *AP1S2* and that miRNA-211 ultimately affected the accumulation of melanin in the skin and coat, which enriches the mechanism of coat color formation and provides theoretical support for coat color regulation.

## Supplement

10.5194/aab-68-125-2025-supplement The supplement related to this article is available online at https://doi.org/10.5194/aab-68-125-2025-supplement.

## Data Availability

The experimental data and the simulation results that support the findings of this study are available on Figshare with the identifier https://doi.org/10.6084/m9.figshare.26312485 (Zhang, 2024).
