# Supplementary material for: The study of miRNA-211 affects melanogenesis progress in Cashmere goats via suppressing AP1S2
Source: Arch Anim Breed. 2025 Feb 11;68(1):125–34. doi: 10.5194/aab-68-125-2025 (PMC13240600; doi:10.5194/aab-68-125-2025)

Supplement of Arch. Anim. Breed., 68, 125–134, 2025
https://doi.org/10.5194/aab-68-125-2025-supplement
© Author(s) 2025. CC BY 4.0 License.

Open Access
Archives Animal Breeding

Supplement of

The study of miRNA-211 affects melanogenesis progress in Cashmere goats via suppressing *AP1S2*

Baoyu Zhang et al.

Correspondence to: Qiaoling Zhang (zql@jlu.edu.cn)

The copyright of individual parts of the supplement might differ from the article licence.

Figure. S1. AP1S2 protein strips. (a) Product AP1S2 (GeneTex) with strips located between 17kDa and 26kDa. (b) The original bands of AP1S2 in vitro transfection of miRNA-211 inhibitors/nc/mimics (three bands on the left) and in black and white Cashmere goats' skin (two bands on the right). (c) The supplementary bands of AP1S2 in vitro transfection of miRNA-211 inhibitors/nc/mimics (d) The supplementary bands of AP1S2 in the skin of black and white Cashmere goats. (e, f) The original bands of AP1S2 were observed in the skin injected anatagomiR-211 and PBS.

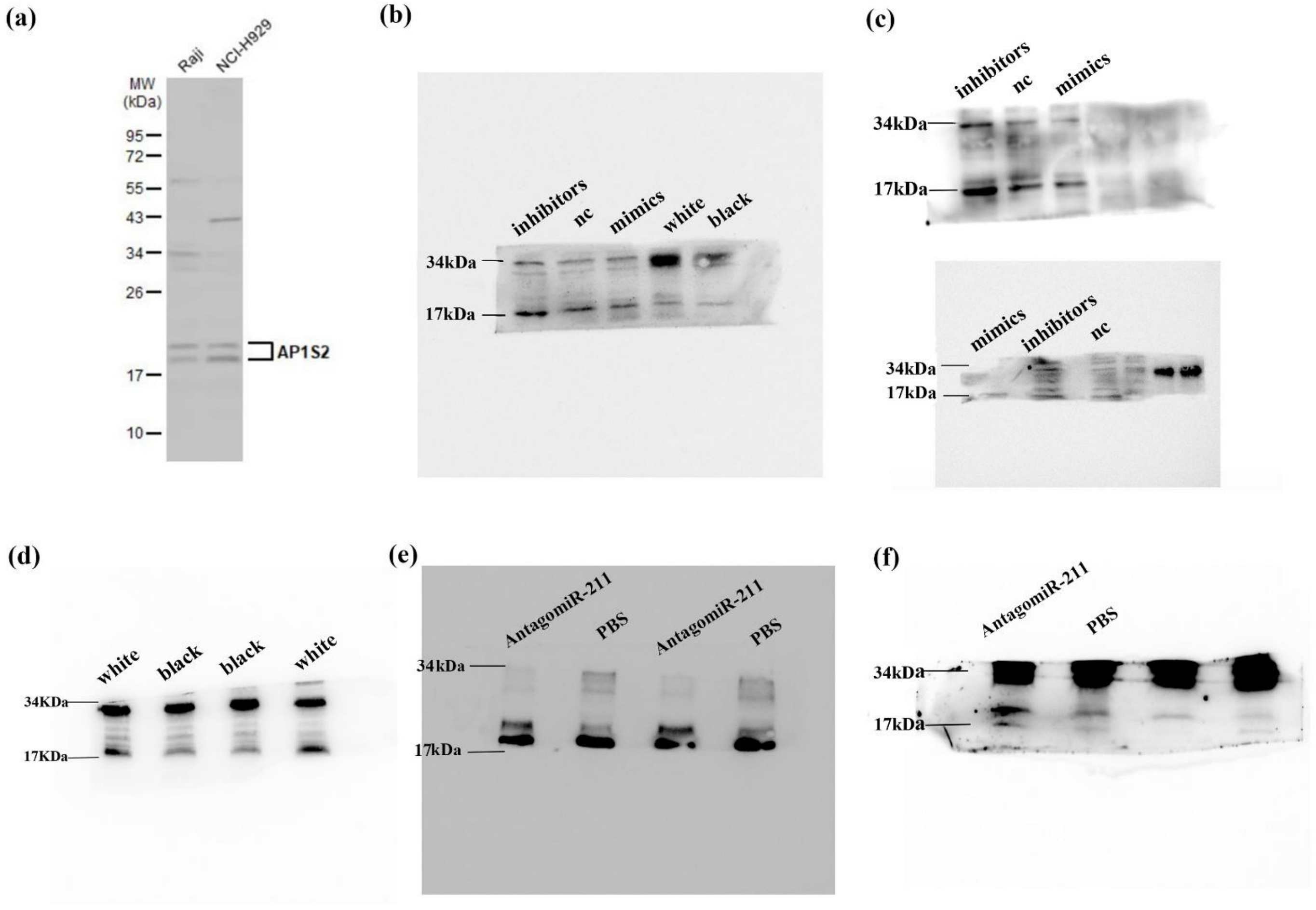

Figure. S2. β-Actin protein strips. (a) Product β-Actin (Proteintech) with strips located about 45kDa. (b) The original bands of β-Actin in miRNA-211 inhibitors/nc/mimics groups (left) and in black and white Cashmere goats' skin (right). (c) The supplementary bands of β-Actin in miRNA-211 inhibitors/nc/mimics groups. (d) The supplementary bands of β-Actin in the skin of black and white Cashmere goats. (e, f) The bands of β-Actin in the AnatagomiR-211 and PBS groups.

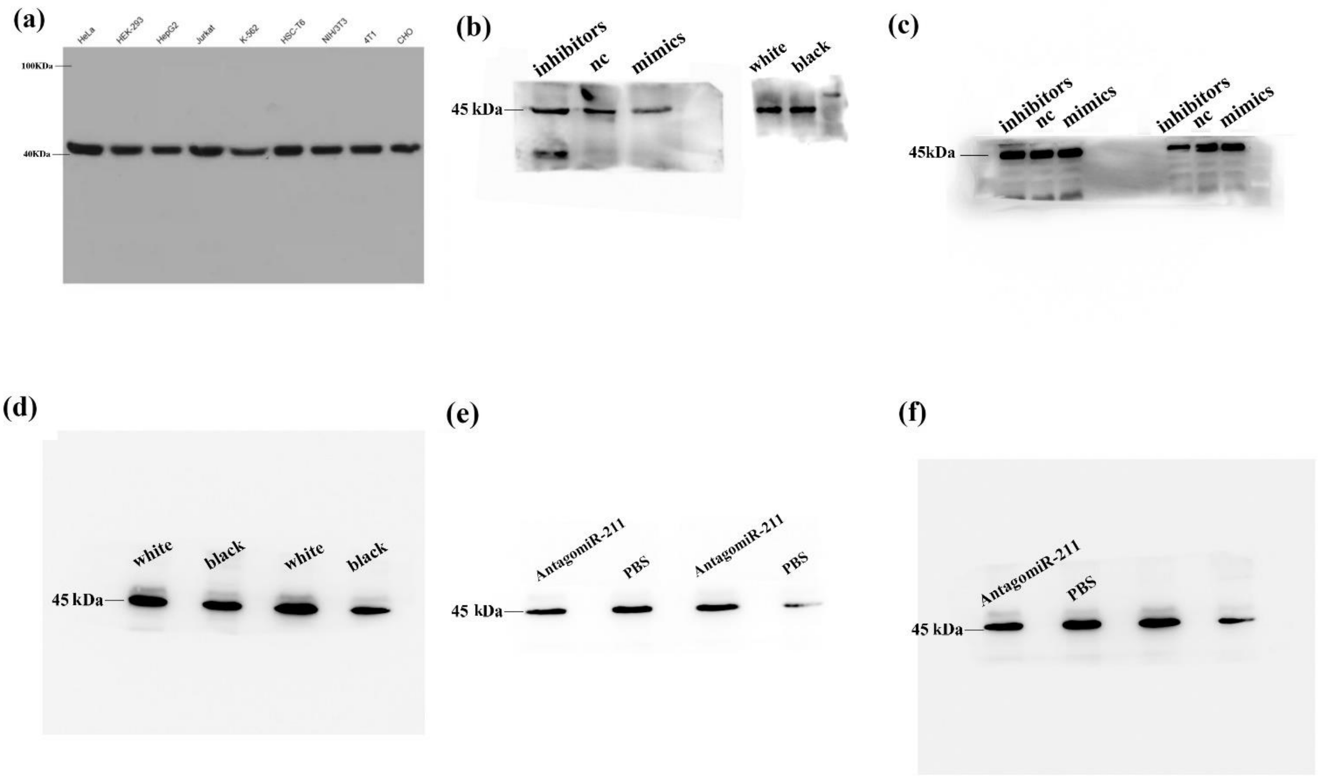

Supplement: The supplement related to this article is available online at https://doi.org/10.5194/aab-68-125-2025-supplement. [file aab-68-125-2025-supplement.pdf]
